# Effect of Nordic Walking and Water Aerobics Training on Body Composition and the Blood Flow in Lower Extremities in Elderly Women

**DOI:** 10.1515/hukin-2015-0012

**Published:** 2015-04-07

**Authors:** Ryszard Jasiński, Małgorzata Socha, Ludmiła Sitko, Katarzyna Kubicka, Marek Woźniewski, Krzysztof A. Sobiech

**Affiliations:** 1 Department of Physiotherapy in Conservative Medicine and Surgery, Faculty of Physiotherapy, University School of Physical Education, Wroclaw, Poland.; 2 Department of Human Biology, Faculty of Physiotherapy, University School of Physical Education, Wroclaw, Poland.

**Keywords:** nordic walking, water aerobic exercise, health training, body composition

## Abstract

Nordic walking and water aerobics are very popular forms of physical activity in the elderly population. The aim of the study was to evaluate the influence of regular health training on the venous blood flow in lower extremities and body composition in women over 50 years old. Twenty-four women of mean age 57.9 (± 3.43) years, randomly divided into three groups (Nordic walking, water aerobics, and non-training), participated in the study. The training lasted 8 weeks, with one-hour sessions twice a week. Dietary habits were not changed. Before and after training vein refilling time and the function of the venous pump of the lower extremities were measured by photoplethysmography. Body composition was determined by bioelectrical impedance. Eight weeks of Nordic walking training improved the venous blood flow in lower extremities and normalized body composition in the direction of reducing chronic venous disorder risk factors. The average values of the refilling time variable (p = 0.04, p = 0.02, respectively) decreased in both the right and the left leg. After training a statistically significant increase in the venous pump function index was found only in the right leg (p = 0.04). A significant increase in fat-free mass, body cell mass and total body water was observed (p = 0.01), whereas body mass, the body mass index, and body fat decreased (p < 0.03). With regard to water aerobic training, no similar changes in the functions of the venous system or body composition were observed.

## Introduction

Chronic venous disorders (CVD) constitute an important social problem and concern approximately 40% of the adult population in Europe and in the United States in spite of introducing new methods of prophylaxis and therapy (Callejas and Manasanch, 2004). Due to the very high incidence of CVD in the adult population, it can be regarded as a social disease of which occurrence may increase along with aging of the population.

Age, sex, family genetic load, sedentary desk work, hard physical work, and obesity are mentioned among CVD risk factors but are not limited to them (Oganov et al., 2006). A higher degree of intensification of CVD clinical symptoms is associated with a shorter refilling time of the veins (RT) and a lower venous pump (VP) capacity (Panny et al., 2009). A fast refilling time of the veins (≤ 20 s) measured by means of photoplethysmography is associated with CVD (Kelechi and Bonham, 2008). Weakness of the calf muscles can contribute to the development of CVD (Padberg et al., 2003). CVD occurs more frequently in women, and their family history, a higher body mass index (BMI) at the age over 45 and low physical activity seem to be related to an increase in the frequency of clinical symptoms (Callejas and Manasanch, 2004).

Moderate physical activity (e.g. walking, cycling, swimming, jogging, dancing, and gymnastics) is of considerable importance in the therapy and prophylaxis of CVD. The deterioration of the function of the musculovascular pump of lower extremities is related to the progression of CVD, whereas an organized physical effort improves the function of calf muscles and venous hemodynamics in ill subjects (Kan and Delis, 2001; Padberg et al., 2004). Obesity is a CVD risk factor that can be modified by physical activity. Obesity and lack of physical activity are more strongly related to CVD in women than in men. Several studies have demonstrated that blood flow parameters differ significantly between healthy individuals with correct body mass and obese individuals, and confirmed the mechanical role of abdominal fatty tissue, excess of which may lead to an increased risk of venous thromboembolism and CVD (Padberg et al., 2003; Willenberg et al., 2010).

Nordic walking (NW) and water aerobics (WA) are recommended for maintaining cardiovascular fitness and for regulation of body composition in the prevention of civilization diseases (Turk et al., 2007; Figard-Fabre et al., 2011; Nuttamonwarakul et al., 2012; Parkatti et al., 2012; Kortmann and Schumacher, 2013).

Exercises in the water are recommended for patients with venous problems. WA covers combined movements of tarsal joints and the calf, which are part of the musculo-vascular pump, and facilitate the outflow of venous blood. The main health effects of WA are increased cardiovascular efficiency, improved metabolism, strengthened muscles with reduced load on joints and normalization of body composition (Barbosa et al., 2009). Regular aerobic exercise in an aquatic environment has a specific influence on fat tissue metabolism. Skeletal muscles obtain free fatty acids as a result of lipolysis. The generated metabolic heat is dissipated faster in water, and more efficiently when its temperature is lower. As the final result, the ratio of muscle tissue to fat tissue (desired body composition) depends on energy expended during the water aerobics, metabolism of fat tissue, thermoregulatory processes and inherited predispositions.

The perception of effort in an aquatic environment is lower than the intensity of performed work. The density of water is greater than air, so exercising in it requires much more intensive effort (Kravitz and Mayo, 1997). Body composition is one of the parameters most often examined in relation to changes which occur in the body under the influence of water training (Barbosa et al., 2009). The studies of the impact of water training on the reduction of fat mass provide contradictory results. Changes were observed ranging from a 4.7% increase to an 11.9% decrease of fat content during training, lasting from 4 to 11 weeks (Kravitz and Mayo, 1997).

NW strengthens most skeletal muscles, including the muscles of lower extremities (Turk et al., 2007), and has a positive impact on body composition in relation to the reduction of risk factors associated with overweight and obesity (Mikalacki et al., 2012). Among numerous benefits resulting from the influence of NW on health, there is a lack of data related to the influence of this kind of activity on the blood flow in venous vessels of lower extremities. The study of Iida et al. (2011) proves that slow walking on a treadmill may increase the blood flow in lower extremities of older women. In subjects with a limited blood flow in lower extremities, slow walking causes muscle hypertrophy and improves muscle strength despite a minimum level of exercise intensity (Abe et al., 2006). NW is recommended as a form of exercise that exerts relatively small loads on the lower extremities. The results of some biomechanical tests indicate that NW does not lead to a decrease of load on the lower limbs in comparison to walking. Thus, the common opinion regarding it should be rejected (Stief et al., 2008).

The aim of this study was to assess the impact of systematic health training, i.e. exercises in the water and Nordic walking, on the venous blood flow in lower extremities and regulation of body composition in women over the age of 50.

## Material and Methods

### Participants

The study was conducted on 24 women aged 51 to 66 (57.9 ± 3.43) years. The assessment of fatness based on the BMI in accordance with the WHO guidelines was as follows: norm 12.5% (BMI<25.0); overweight 66.7% (25≤BMI<30.0); obesity 20.8% (BMI≥30). The group was randomly divided into three subgroups of eight subjects each: G1 – women taking part in NW training, G2 – women taking part in WA classes, and G3 – women who did not take part in any forms of physical activity. Anthropometric, blood flow and body composition measurements were performed prior to the beginning and after 8 weeks of the NW and WA training, under identical conditions of measurements and always at the same time of the day. For G3, the measurements were carried out at the beginning and after completion of the research project. The subjects were instructed neither to change their diet during the training program nor to introduce other types of physical exercise. Participation in the study was voluntary and required signing a consent form. The study was approved by the Bioethics Committee of the Medical University in Wroclaw, no. KB-258/20143, dated 13.06.2013.

### Measures

The venous blood flow of lower extremities was examined using the photoplethysmography method with a Rheo Dopplex II PPG device manufactured by Huntleigh Diagnostics, England. Vein refilling time (RT) and operation of the lower extremity venous pump (VP) were measured. The individual examined was seated, lower extremities bent at an angle of about 110 degrees at the knee and feet placed flat on the floor. The photoplethysmography sensor was fixed at the level of approximately 10 cm above and slightly to the rear of the medial ankle. The examined person performed 10 rhythmic movements of dorsal feet flexions (blood outflow), then remained immobile for 45 s (vein filling with blood). The device registered VP and RT parameters from the first feet flexion movement. First, a curve was obtained illustrating blood pumping from the feet and lower leg areas, and then a slow decrease describing the time of blood return to vascular bed of the lower extremity. In healthy subjects, the RT parameter standard in the examination of a lower extremity should be ≥ 45 s, whereas the VP parameter should be ≥ 40 s. The measurements were performed three times, separately for the right and left leg, and then an average value was calculated.

Anthropometric techniques as well as bioelectric impedance analysis (BIA) (STA/BIA RJL analyzer – Akern 101/S tetrapolar version, Italy) were used to assess body composition. This evaluation was performed after an overnight fast. Body mass and height were measured and the value of body mass index (BMI) was calculated [body mass (kg) / body height (m^2^)]. The following components of body composition, according to BIA, were considered: total body water (TBW) [%], body fat (BF) [%], fat-free mass (FFM) [%], and body cell mass (BCM) [%].

### Procedures

An eight-week NW training cycle was conducted. The classes were held twice a week and lasted one hour at an intensity of 4.8 MET (metabolic equivalents) on the basis of the Compendium of Physical Activities according to Ainsworth et al. (2011). They were always carried out at the same pace along the same route. A single session consisted of the following parts: initial part – a 10 min warm-up, main part – walking for 40 min, final part (cool down) – 10 min. Uniform sticks manufactured by KV2 (Switzerland) were used for the classes; the length of sticks was adjusted for each person individually according to body height of each subject. Training was conducted by two physiotherapists: an NW coach and an NW instructor. It covered walking in accordance with the NW step methodology at an elementary level. Before, during and after each training unit, the trainers checked the general feeling of the seniors using a simplified 10-degree scale of tiredness and breathlessness according to Borg, and, if necessary, some of the women were excluded from specific exercises.

The WA training lasted eight weeks (twice a week, 45 minutes each session). The intensity of exercises was defined as light (3.2–5.3 MET) according to Raffaelli et al. (2012). The exercises were conducted in the water at a temperature of 31–32°C at a depth of 1.4 m (a deep water aerobic program). During training, accessories that increased water resistance were used, e.g. flotation belts. Each session consisted of three parts: pre-class instructions – the instructor’s remarks on safety; a warm up – exercises that accustomed subjects to the water, its temperature and depth, increased body temperature, and prepared joints and muscles for exercise; and the main part – aerobic exercises (cardio) and exercises that strengthened muscles. The aim of the main part was to strengthen the cardiovascular system and the muscular system through several repetitions with proper intensity controlled by the rhythm of music. The final part (cool down – an active rest) consisted of slow movements of the extremities of relaxing character with elements of respiratory exercises.

### Statistical analysis

Statistical calculations were performed using the Statistica 9.0 program. The differences between two samples of dependent variables (before and after the training) were examined using the Wilcoxon signed-rank test. For the comparison of 3 independent groups (G1, G2 and G3 in the preliminary examination) the Kruskal-Wallis ANOVA test was applied. A significance level of p<0.05 was adopted.

## Results

In the preliminary examination, the difference between groups G1, G2, and G3 was not statistically significant in relation to age, body mass, height, the BMI or body composition components (Table 1).

Similarly, the comparison of average values of the RT and VP blood flow parameters performed on the examined groups G1, G2, and the control group G3 at the baseline showed no statistically significant differences. For all groups, the average value of the aforementioned parameters was lower than the recommended value and proved the occurrence of CVD (Table 2).

In women from group G1, body mass, the BMI and BF% decreased (p = 0.03, p = 0.03, p = 0.01, respectively), and the content of FFM%, TBW%, and BCM% increased statistically significantly (p = 0.01, respectively) compared to baseline values and after 8 weeks of NW training (Table 1). Eight-week WA training did not have a significant impact on anthropometric variables or body composition. In the G2 group a statistically significant decrease of BCM% was observed (p = 0.02). In the non-training group of women G3, no significant changes in anthropometric and body composition indices were found between the preliminary and final examination. Changes of blood flow parameters in the lower extremities occurred only in group G1 after 8 weeks of NW training (Table 2). In G1, the average values of the RT parameter (p = 0.04, p = 0.02, respectively) decreased both in the right and left lower extremity. Compared to baseline values, the effectiveness of the venous pump of lower extremities measured with the VP parameter improved, which was statistically significant for the right extremity (p = 0.04). In group G2, which followed the WA training program, no significant changes in blood flow parameters were observed. Similarly, no significant changes in blood flow parameters occurred in the non-training group (G3) between the preliminary and final examination.

## Discussion

NW and WA are forms of physical activity that are recently very popular among the population of older people. The results of many studies confirm the significance of physical effort for reducing the risk of vascular diseases and diseases related to body composition disorders (Padberg et al., 2004; Lee et al., 2012). This paper attempts to evaluate the impact of regular eight-week NW and WA training in relation to the function of the venous system of lower extremities and body mass regulation in the context of reduction of the risk factors associated with excess BF.

Medical history and the results of the photoplethysmography examination indicated an existing problem in the vascular system of the middle aged women included in the study. The applied non-invasive photoplethysmography method is a technique fully approved in clinical conditions for the purpose of screening tests as part of the assessment of the lower extremity veins (Kelechi and Bonham, 2008). A lower value of the RP parameter in lower extremities compared to its standard value may indicate some abnormalities of the functions of venous valves. However, a low value of the VP parameter results from the general physical condition of the subjects, small muscle mass, lack of physical activity and general circulation problems related to both the venous system and the arterial system. A reduction of the RT parameter in the standard test may indicate a valvular insufficiency or venous reflux. The value of RT at the level of 15 s should be an indication for performing a specialized medical test. Regarding the analyzed group of women who took part in NW training, a reduction of the time needed for the inflow of blood from the foot and lower leg areas after its pumping out was noted. It is likely to be associated with improvement of the condition of the blood supply to the lower extremities caused by regular physical effort such as walking (Lejczak et al., 2011). Such effort enhances blood circulation of a lower extremity. Activation of a greater number of arterial blood inflows occurs, which results in a faster blood flow (Morgulec-Adamowicz et al., 2011). Such an assumption is confirmed by an increase in the value of the VP parameter that informs about work of the musculo-vascular pump. In the present study, a statistically significant increase in the value of the VP parameter occurred in both lower extremities only in the NW group, indicating pumping of a considerably greater volume of blood in the course of the final examination when compared to the examination performed before the training program started. Some authors indicate distinctly greater effectiveness of NW training for improving blood circulation compared to other types of regular physical activity (Purzycka et al., 2011; Parkatti et al., 2012).

NW is a physical activity based mainly on walking (Abe et al., 2006; Stief et al., 2008; Turk et al., 2007). During the walk, appropriate muscle groups work by contracting, thus influencing blood vessels and causing evacuation of blood from vein vessels. In addition, muscle contraction prevents blood from being retained in peripheral blood vessels. Training of appropriate muscles (typical for the muscle pump) improves the intramuscular coordination and causes a better blood flow, increased venous tonus and less retention of blood in the lower extremities. Deep veins are responsible for 80–90% of the venous return. Blood contained in their lumen is subjected to three forces driving it towards the heart. These are, successively: the sucking effect of the abdomino-thoracic pump and right heart (vis a fronte); the force of pumping blood flowing from the capillaries (vis a tergo); and an extremely important force compressing the vein from outside once the muscle adjacent to the vein has contracted in a confined subfascial space (vis a latere). The latter factor is responsible for at least 30% of the force driving blood in the veins. So there is no exaggeration in the terms “muscle pump” and “peripheral heart”. An additional force is the pulsation of the adjacent artery. In small vessels the pressure transfer is facilitated by the common connective tissue sheath of the vascular bundle. In the iliac section, veins run between homonymous arteries and the ilium and sacrum, which also creates conditions to transfer the wave of heightened blood pressure in the vein lumen (Kan and Delis, 2001; Padberg et al., 2004).

According to various studies (e.g. Barbosa et al., 2009), WA reduces muscle tension. This may influence blood retention in peripheral sections of vein vessels or cause a slowed down blood flow in vessels. The final result is a reduction of the RT parameter. In the process of blood rheography in lower extremities, functioning of the musculovascular pump is of great importance. Rhythmical contraction of the calf muscle triggers the mechanism of this pump. The best conditions for proper functioning of the musculo-vascular pump are delivered by the walking movements that dominate NW training. For a proper venous flow, a well-functioning valvular apparatus is necessary that prevents pendular movement of blood or even a pathological flow to peripheral areas (reflux). Appropriate valves divide the blood column in a vessel into isolated sections. As a result of the above-mentioned forces, the hydrostatic pressure in those sections periodically increases or decreases. The hydrostatic pressure in aqueous conditions increases depending on the volume of water in which the exercising person is immersed, immersion depth and his/her position. The pressure difference between water and the human body influences the distribution of blood masses and functioning of skeletal muscles. The increased hydrostatic pressure may hinder the above-described mechanisms of the venous blood outflow from lower extremities. This may influence both functioning of the “muscle pump” and the valves. Not without significance is the position in which the exercises are performed; it facilitates blood retention in distal sections of the extremities that with increased resistance may further impede a venous return (Kan and Delis, 2001; Kravitz and Mayo, 2004; Padberg et al., 2004; Barbosa et al., 2009).

Overweight and obesity, which occurred in 87.5% of the examined women, may be among the causes of vascular changes. A relationship between the BMI and CVD has been observed (Jawień et al., 2003), particularly in women, although it is not always confirmed (Kanchanabat et al., 2010). The compression of veins in the abdominal cavity resulting from an increased amount of extraperitoneal fat may lead to an increase of venous blood pressure and the development of varicose veins (Sudoł-Szopińska et al., 2006).

For the treatment of patients with excess body mass, aerobic exercises of moderate intensity are recommended. The recommended forms of physical activity are as follows: NW, WA, cycling and resistance training (Kortmann and Schumacher, 2013). In the present study the women performing the health training program did not use any prescribed reductive diet. The exercises performed within the program were the only regular physical activity. Benefits resulting from the participation in the eight-week training program, in relation to the regulation of body mass and the improvement of venous flows in the lower extremities, occurred only in the women who performed NW training. Out of the CVD risk factors, body mass, the BMI, and BF statistically significantly decreased as a result of NW training, whereas the content of tissue components, indicating the development of muscle mass, FFM, TBW, and BCM, increased. Similar changes of body mass, the BMI and BF in women at menopausal age with excess fatty tissue, including patients with diabetes type 2, after 12 weeks of NW were observed by other authors (Gram et al., 2010; Figard-Fabre et al., 2011). Improvement of average values of anthropometric measurements and fitness in even older overweight subjects (60 ± 5.3 years) with glucose tolerance disorders after 16 weeks of NW training was observed by Fritz et al. (2013). Compared to the above-mentioned research results, our observations indicate that the improvement of body composition variables towards reduction of risk factors related to overweight and obesity in women at menopausal age can be expected already after eight weeks of regular NW training.

In the WA group, 8 weeks of training with a frequency of twice a week and each training session lasting 45 min were not enough to evoke significant changes in body composition associated with the reduction of BF and an increase in FFM and BCM. Śliwicka et al. (2007) obtained similar results in a group of overweight and obese middle-aged women who took part in WA classes for a period of 12 weeks (twice a week, each class lasting 45 min). In the group of women examined by the above-mentioned authors, no statistically significant changes were found related to body mass and the BMI; however, their glucose tolerance improved due to regular physical activity. Similarly, a 12-week NW program (3 times a week, each class lasting 60 min) did not cause a reduction of body mass or the BMI, in women (63 years of age) with type 2 diabetes (Reis Filho et al., 2012). Reduction of body mass and %BF but no change of the BMI value and an increase in muscle strength were observed by Nuttamonwarakul et al. (2012) in patients with diabetes, aged over 60 years, who participated in 12 weeks of WA training, 3 times a week, each class lasting 50 min. However, this study did not include the division of subjects into sexes. Our results confirm the opinion of Kravitz and Mayo (2004) as well as Barbosa et al. (2009). The positive effects in fat tissue reduction can only be achieved after a minimum of 8 weeks of WA with a simultaneous dietary restriction, whereas without such a diet the training program should last longer than 8 weeks.

Our study should be treated as preliminary, and at this moment the results cannot be generalized. Major limitations of this work are the small sample size and the relatively short training period. At this stage of research it was found that even a relatively short period of NW training causes expected changes in body composition and affects the blood flow in lower extremities. Our further experiments evaluating the effect of WA on the vascular system and body composition will take into account an increase in duration of training up to 12 weeks. The effects of WA and NW training will be analyzed separately in groups of normal weight, overweight and obese women.

A combination of systematic NW or WA physical exercise and maintaining proper body mass can improve the process of rehabilitation in vascular diseases.

## Conclusion

Eight weeks of NW training in women aged over 50 years improve the venous blood flow in lower extremities and normalize body composition towards the reduction of CVD risk factors.

Eight weeks of WA training in women aged over 50 years are not sufficient to significantly change body composition towards a reduction of body fat content and development of muscle mass and do not lead to an improvement of the function of the musculo-vascular pump of lower extremities.

## Figures and Tables

**Table 1:** The effects of different exercise programs on anthropometric features and body composition variables

Variables	Group									

	G1: Nordic walking (N=8)		*p*^a^	G2: Water aerobics (N=8)		*p*^a^	G3: Non-exercising (N=8)		*p*^a^	*p*^b^
		
	Baseline	Eight weeks		Baseline	Eight weeks		Baseline	Eight weeks		
Age (years)	57.9 ± 1.64			57.3 ± 4.83			58.7 ± 3.38			0.56
Body height (cm)	165.3 ± 6.22			164.6 ± 4.94			161.4 ± 6.29			0.33
Body mass (kg)	74.8 ± 5.82	73.8 ± 6.18	0.03^*^	73.7 ± 8.98	76.0 ± 7.82	0.12	71.3 ± 5.24	71.6 ± 6.06	0.80	0.53
BMI (kg/m^2^)	27.3 ± 1.54	27.0 ± 1.40	0.03^*^	27.8 ± 2.93	28.1 ± 2.97	0.12	27.5 ± 3.02	27.6 ± 3.41	0.74	0.77
TBW (%)	45.5 ± 3.19	47.9 ± 2.71	0.01^*^	46.9 ± 3.02	46.8 ± 4.41	0.78	47.7 ± 2.44	47.5 ± 2.43	0.58	0.60
BF (%)	35.1 ± 3.19	32.6 ± 2.76	0.01^*^	33.8 ± 2.76	34.3 ± 2.73	0.67	33.4 ± 2.94	33.6 ± 2.97	0.58	0.60
FFM (%)	65.0 ± 3.19	67.4 ± 2.76	0.01^*^	66.2 ± 2.76	65.7 ± 2.73	0.61	66.6 ± 2.94	66.4 ± 2.97	0.58	0.36
BCM (%)	33.6 ± 1.59	36.2 ± 1.38	0.01^*^	36.2 ± 2.84	33.6 ± 2.50	0.02^*^	35.0 ± 3.00	35.1 ± 2.85	0.72	0.09

BIA (body impedance method), BMI (body mass index), BF (body fat), FFM (fat free mass), TBW (total body water), BCM (body cell mass). Values are mean ± SD.

a Wilcoxon signed-rank test to compare two dependent variables, for comparison between baseline and after eight weeks of training.

b nonparametric ANOVA Kruscal-Wallis test, to compare many independent trials, for comparison between baseline G1, G2 and G3 groups.

* p < 0.05.

**Table 2:** The effects of different exercise programs on blood flow parameters of the lower limbs

	Group									

Variables	G1: Nordic walking (N=8)		*p*^a^	G2: Water aerobics (N=8)		*p*^a^	G3: Non-exercising (N=8)		*p*^a^	*p*^b^
		
	Baseline	Eight weeks		Baseline	Eight weeks		Baseline	Eight weeks		
RT right (s)	39.1 ± 10.83	31.0 ± 10.45	0.04^*^	32.3 ± 16.82	33.6 ± 15.10	0.11	25.3 ± 9.30	24.9 ± 9.03	0.40	0.08
RT left (s)	40.8 ± 5.68	32.1 ± 9.75	0.02^*^	29.0 ± 11.01	31.5 ± 10.93	0.08	37.4 ± 7.93	37.0 ± 5.50	0.48	0.07
VP right	29.4 ± 6.44	36.1 ± 10.27	0.04^*^	25.6 ± 10.34	31.1 ± 8.41	0.09	37.3 ± 12.06	38.9 ± 12.37	0.89	0.24
VP left	34.6 ± 9.10	39.0 ± 6.85	0.12	38.1 ± 15.13	35.0 ± 11.50	0.09	34.5 ± 8.96	33.3 ± 9.10	0.48	0.83

RT (refiling time), VP (venous pump). Values are mean ± SD.

a Wilcoxon signed-rank test to compare two dependent variables, for comparison between baseline and after eight weeks of training.

b nonparametric ANOVA Kruskal-Wallis test, to compare many independent trials, for comparison between baseline G1, G2 and G3 groups.

* p < 0.05.
